# AgIn_5_S_8_/g-C_3_N_4_ Composite Photocatalyst Coupled with Low-Temperature Plasma-Enhanced Degradation of Hydroxypropyl-Guar-Simulated Oilfield Wastewater

**DOI:** 10.3390/molecules29122862

**Published:** 2024-06-16

**Authors:** Xiang Li, Yuhang Zhang, Yiling Wang, Li Zhu, Yuhang Liu, Lingxing Wang

**Affiliations:** 1 School of Civil Engineering and Architecture, Chongqing University of Science and Technology, Chongqing 401331, China; m19827351027@163.com (Y.Z.); setempty2023@163.com (Y.W.); m15111875386@163.com (L.Z.); 2020442981@cqust.edu.cn (Y.L.); 2020441991@cqust.edu.cn (L.W.); 2 Key Laboratory of the Three Gorges Reservoir Regions Eco-Environment, State Ministry of Education, Chongqing University, Chongqing 400045, China

**Keywords:** dielectric-barrier discharge–low-temperature plasma (DBD–LTP), photocatalyst, oilfield wastewater, graphitic carbon nitride

## Abstract

The effective treatment and recovery of fracturing wastewater has always been one of the difficult problems to be solved in oilfield wastewater treatment. Accordingly, in this paper, photocatalytic-coupled low-temperature plasma technology was used to degrade the simulated wastewater containing hydroxypropyl guar, the main component of fracturing fluid. Results indicated that hydroxypropyl-guar wastewater could be degraded to a certain extent by either photocatalytic technology or plasma technology; the chemical oxygen demand and viscosity of the treated wastewater under two single-technique optimal conditions were 781 mg·L^−1^, 0.79 mPa·s^−1^ and 1296 mg·L^−1^, 1.01 mPa·s^−1^, respectively. Furthermore, the effective coupling of AgIn_5_S_8_/gC_3_N_4_ photocatalysis and dielectric-barrier discharge–low-temperature plasma not only enhanced the degradation degree of hydroxypropyl guar but also improved its degradation efficiency. Under the optimal conditions of coupling treatment, the hydroxypropyl-guar wastewater achieved the effect of a single treatment within 6 min, and the chemical oxygen demand and viscosity of the treated wastewater reduced to below 490 mg·L^−1^ and 0.65 mPa·s^−1^, respectively. In the process of coupled treatment, the AgIn_5_S_8_/gC_3_N_4_ could directly absorb the light and strong electric field generated by the system discharge and play an important role in the photocatalytic degradation, thus effectively improving the energy utilization rate of the discharge system and enhancing the degradation efficiency of hydroxypropyl guar.

## 1. Introduction

Petroleum is a strategic resource of a country and is non-renewable. The exploitation of low-permeability oilfields is the focus of oil exploitation. Therefore, in order to develop low-permeability oil and gas fields with high efficiency and low cost, techniques such as increasing production and injection by fracturing in oil wells and repeated acidification are often adopted [1,2,3]. Among them, fracturing stimulation technology has become the main measure for the production of such oil and gas fields. The wastewater produced after oilfield fracturing operations has the characteristics of high turbidity, high viscosity, and high chemical oxygen demand (COD), which is the most difficult wastewater in oilfield wastewater treatment [4,5,6,7]. This kind of wastewater is large and highly dispersed, and if not treated effectively, it will cause serious pollution to the environment. How to effectively treat and recycle oilfield fracturing wastewater while maintaining energy conservation and emission reduction, environmental protection and water resources protection is one of the urgent problems to be solved at present. Therefore, it is of great practical significance to develop low permeability oil and gas fields with high efficiency and low cost to carry out the research on fracturing wastewater treatment technology suitable for the current situation of fracturing operations in oil and gas fields.

Oilfield fracturing wastewater is characterized by a large amount of water, complex and stable organic composition, and difficult to degrade [8,9,10]. The high viscosity and high COD_Cr_ characteristics of oilfield fracturing wastewater are both derived from the large number of polymer hydroxypropyl-guar compounds contained in the wastewater. In theory, if the chain of hydroxypropyl-guar molecules in the wastewater can be broken up to degrade it into small molecular substances, the viscosity of the wastewater would be greatly reduced and can improve the biochemical properties of wastewater to reduce the load of subsequent treatments. However, the research on its degradation technology in the world is still in the initial stage. The treatment effect of traditional chemical and physical methods is not ideal. If the membrane processing method is used, the cost is high and the requirements for equipment operation and maintenance are also relatively high [10,11,12,13]. In addition, the existing process always has some restrictive disadvantages, or the COD_cr_ and viscosity of the degraded wastewater still make it difficult to meet the requirements of the subsequent biological treatment, or the treatment time and cost are high. Therefore, the study of oilfield fracturing wastewater treatment technology has become a focus of many experts, scholars, and engineers.

Photocatalytic technology is an advanced water treatment technology that has been used in recent years. It is a simple process that has the advantages of effectively damaging the structure of organic pollutants, low energy consumption and raw material consumption, and does not cause damage to the background environment or secondary pollution [14,15,16,17,18,19,20]. Therefore, it is a promising environmentally friendly water treatment method. In recent years, photocatalytic technology is mainly used in the degradation of printing and dyeing wastewater [21,22,23], papermaking wastewater [24,25], pharmaceutical wastewater [26], and other fields. Recent studies have shown that photocatalytic technology has great potential in the field of oily wastewater treatment [27,28,29]. Therefore, researchers began to focus on photocatalytic treatment of oily wastewater. However, the conventional photocatalyst treatment of oilfield fracturing wastewater still cannot achieve the desired effect. The development of new photocatalysts for selective and efficient treatment of oilfield fracturing wastewater or the use of some coupling technologies to achieve the pretreatment effect is a difficulty in this field.

Low-temperature plasma technology is a new advanced oxidation technology in the field of international environmental engineering in recent years [30,31,32]. Unlike the traditional advanced oxidation technology, low-temperature plasma technology can produce many high-energy particles with super-chemical activity (electrons, ions, ·OH and other free radicals, ultraviolet light, etc.) through plasma discharge. These high-energy particles can stimulate the activity of neutral particles, so as to force various high-energy organic bonds (such as C-C, C=O, C=C) to break, and finally reduce the molecular weight of high molecular organic matter and the viscosity of high molecular organic wastewater [31,32,33]. In view of the characteristics of low-temperature plasma and its various characteristics in the degradation of organic compounds, it can be found that due to the limitations of technical conditions, it cannot be used as an independent technology for the degradation of high concentration polymer organic wastewater. However, it can break the macromolecular chain, reduce the viscosity of polymer solution, and thus, it has great potential to reduce the difficulty of chemical and biochemical treatment of refractory organic compounds.

In view of the difficult degradation characteristics of oilfield fracturing wastewater, this paper carried out a study on the combined treatment process, combining photocatalytic technology with low-temperature plasma technology to strengthen the degradation of polymer organic components in fracturing wastewater, so as to effectively improve the biodegradability of wastewater. In the process of low-temperature plasma treatment of wastewater, in addition to the effective degradation chemical reaction caused by active substances in the system, the physical effects such as light and the electric field produced by the discharge can also induce the chemical reaction process. In addition, in order to improve the energy utilization rate of the discharge system, it is a very reasonable and effective method to improve the efficiency of light and the electric field. Studies have shown that UV light and a strong electric field generated by the plasma discharge can stimulate photocatalytic materials. Therefore, the introduction of nano-composite photocatalytic materials in this process can directly absorb the light and strong electric field generated by the discharge of the system to have a photocatalytic degradation effect, so as to effectively improve the energy utilization rate of the discharge system and enhance the degradation efficiency of polymer organic matter.

## 2. Results and Discussion

### 2.1. Photocatalytic Activity of AgIn_5_S_8_/g-C_3_N_4_ for Hydroxypropyl-Guar Wastewater

**Figure 1:**
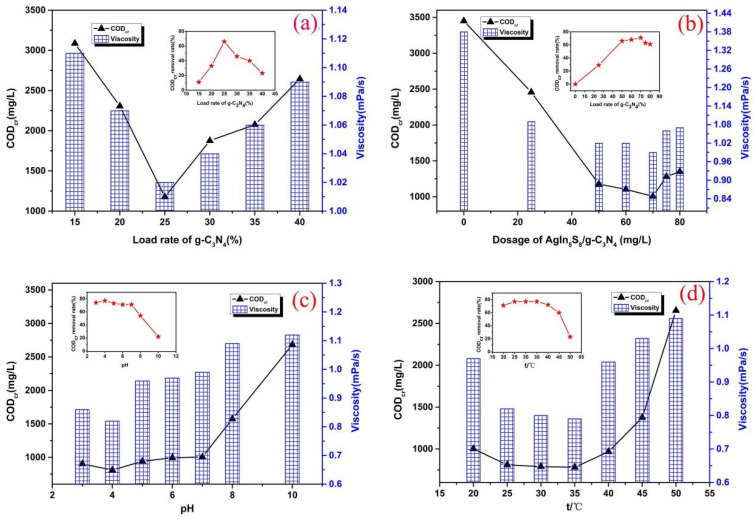
Effect of load rate of g-C_3_N_4_ (**a**), dosage of AgIn_5_S_8_/g-C_3_N_4_ (**b**), pH (**c**), and t (**d**) on the photocatalytic treatment of hydroxypropyl-guar wastewater by AgIn_5_S_8_/g-C_3_N_4_.

The experimental results of the major factors affecting AgIn_5_S_8_/g-C_3_N_4_ photocatalytic degradation of hydroxypropyl-guar wastewater are shown in Figure 1. It is clear that the load rate of g-C_3_N_4_ on the photocatalyst is one of the main factors affecting its photocatalytic properties. With an increase in the g-C_3_N_4_ load rate (5~25%), the photocatalytic effect of AgIn_5_S_8_/g-C_3_N_4_ on hydroxypropyl guar increased significantly, and when the g-C_3_N_4_ load rate exceeded 25% and continued to increase, the photocatalytic performance of the material was reduced, manifested in the decrease in the COD removal rate of the treated water samples (Figure 1a: 50 mg/L of AgIn_5_S_8_/g-C_3_N_4_ dosage, 7 of pH, 10 min of treatment time, 25 °C). The deteriorating photocatalytic performance of the 35% g-C_3_N_4_ load rate can be attributed to the overload of g-C_3_N_4_ covering the entire surface of AgIn_5_S_8_, resulting in poor AgIn_5_S_8_ photosensitizer collection and also reducing the active site on the AgIn_5_S_8_ surface. In addition, the AgIn_5_S_8_ nanoparticles would also cluster together under light exposure. Therefore, composite photocatalysts with a 25% g-C_3_N_4_ load rate have the best catalytic properties. Meanwhile, the above results also clearly indicate that the synergistic effect between AgIn_5_S_8_ and g-C_3_N_4_ played an important role in the photocatalytic process.

The dosage of the photocatalyst would affect the number of oxidation activity factors. It was necessary to study, under the same conditions, its effect on the degradation of hydroxypropyl guar (Figure 1b: 25% of g-C_3_N_4_ load rate, 7 of pH, 10 min of treatment time, 25 °C). When the AgIn_5_S_8_/g-C_3_N_4_ dosage was 70 mg/L, the best degradation of hydroxypropyl guar was obtained. When the dosage of the photocatalyst was less than 70 mg/L, less photocatalyst produced insufficient active species, and the reduction effect of pollutants was not optimal. Conversely, when the dosage of the photocatalyst exceeded 70 mg/L, excessive photocatalyst powder would increase the solid phase content of the suspension, so that the visible light was blocked by excessive catalyst [34,35,36]. Therefore, the process of photon delivery to the surface of the photocatalyst powder was affected, preventing the production of the active species, and thus causing a decrease in the efficiency of the photocatalytic degradation of hydroxypropyl guar.

**Figure 2:**
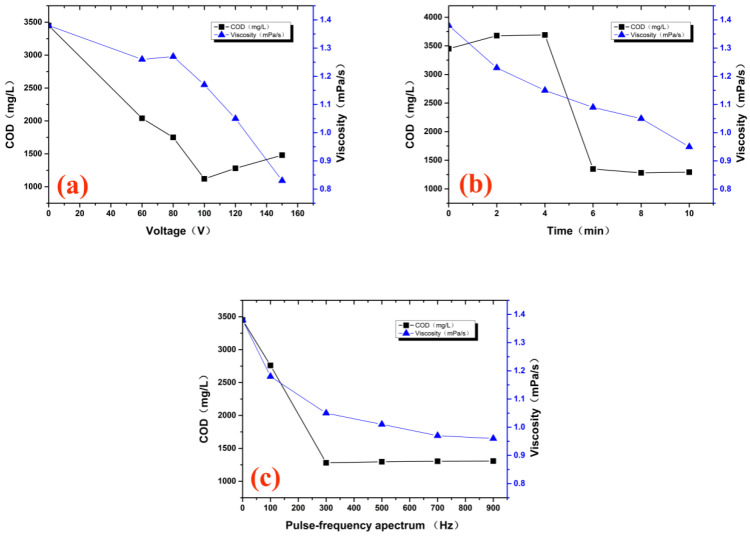
Effect of voltage (**a**), discharge time (**b**), and pulse frequency (**c**) on the hydroxypropyl-guar degradation by DBD–LTP.

It has been shown that the solution pH would affect the degradation effect of the photocatalyst on organic matter, so it was necessary to evaluate the effect of pH on the degradation of hydroxypropyl guar by AgIn_5_S_8_/g-C_3_N_4_ (Figure 2c: 25% of g-C_3_N_4_ load rate, 70 mg/L of AgIn_5_S_8_/g-C_3_N_4_ dosage, 10 min of treatment time, 25 °C). As shown in Figure 2c, in the process of increasing the solution pH from 3 to 10, the degradation effect of the photocatalyst for hydroxypropyl guar was first increased and then reduced, and the optimal solution pH was 4. The pH of the solution could affect the charge distribution on the surface of the composite photocatalyst, and there was a large amount of H^+^ in the weakly acidic aqueous solution, which was conducive to the formation of reactive oxygen species [37,38]. 

As can be seen from Figure 1d (25% of g-C_3_N_4_ load rate, 70 mg/L of AgIn_5_S_8_/g-C_3_N_4_ dosage, 4 of pH, 10 min of treatment time), the solution temperature had a great impact on the degradation of hydroxypropyl guar by the AgIn_5_S_8_/g-C_3_N_4_. As the solution temperature gradually increased from 20 °C to 50 °C, the degradation effect on hydroxypropyl guar increased first and then decreased. An increase in the temperature can improve the movement rate of ions and groups in the solution, and can significantly improve the catalytic reaction rate, so the appropriate temperature increase can better help the photocatalyst to degrade hydroxypropyl guar. The antiregular results of the catalytic degradation effect over 35 °C should come from two aspects. On the one hand, the excessive increase in temperature would affect the self-decomposition of AgIn5S8. On the other hand, the hydroxypropyl-guar solution would evaporate a part of the water with an increase in the solution temperature, indirectly resulting in an increase in the hydroxypropyl-guar concentration, which was not conducive to the improvement of degradation. The optimum temperature, shown in Figure 1d, was 35 °C. However, the degradation effect at 25~35 °C was seen to be a little different. If only photocatalytic technology is used to degrade hydroxypropyl guar, the temperature of 25 °C is more appropriate. 

### 2.2. Treatment of Hydroxypropyl-Guar Wastewater by DBD–LTP

The effects of different voltages on the COD and viscosity of treated wastewater are shown in Figure 2a (discharge time: 8 min; ventilation: 12 L/min; pulse frequency: 300 Hz). It can be seen from the figure that when other treatment conditions were unchanged and only the voltage was changed, the COD value of the treated wastewater decreased first and then increased, while the viscosity of the wastewater decreased gradually. The reason for this phenomenon may be that some easily degraded substances could be degraded under low voltage, while the refractory substances cannot be degraded or oxidized by potassium dichromate under low voltage, so the measured COD value was lower than the actual value. When the voltage increases, the part of the substance that cannot be degraded under low voltage conditions is further degraded and can be oxidized by potassium dichromate, so the COD value measured at this time increases. With an increase in the voltage, the organic matter in the solution is continuously degraded into low molecular substances, so the viscosity is continuously decreased. Under this experimental condition, the COD value and viscosity of the treated hydroxypropyl-guar wastewater were 1280 mg/L and 1.05 mPa/s, respectively. Considering the energy consumption, the processing voltage used in the subsequent experiments was 120 V.

The influence of different discharge times on the COD and viscosity of treated wastewater is shown in Figure 2b (voltage: 120 V; ventilation: 12 L/min; pulse frequency: 300 Hz). It can be seen from the figure that when the other treatment conditions remain unchanged and only the discharge time is changed, the COD value of the treated wastewater also shows a trend of first increasing and then decreasing. The reason for the increase in the COD in the initial stage of the experiment is that hydroxypropyl guar is a refractory organic matter. In the process of discharge, the structure of hydroxypropyl guar is destroyed, and the macromolecular organic matter is broken to form small molecular organic matter, so that the refractory groups that cannot be oxidized by potassium dichromate are degraded into substances that can be oxidized by potassium dichromate after plasma treatment, which leads to an increase in the COD. Due to the increase in active substances in the plasma system, and the refractory groups in the wastewater breaking into small molecular substances, with the discharge, the small molecular organic matter in the wastewater is degraded into water and carbon dioxide, so the COD of the wastewater continues to decline. As the discharge goes on, the concentration of macromolecules decreases, so the viscosity decreases. Also, considering the processing economy, the discharge time is 8 min.

The effects of different pulse frequencies on the COD and viscosity of treated wastewater are shown in Figure 2c (voltage: 120 V; ventilation: 12 L/min; discharge time: 8 min). It can be seen from the figure that when other treatment conditions remain unchanged and only the pulse frequency is changed, the COD value and viscosity of the treated wastewater decrease continuously with the increase in the discharge frequency. When the pulse frequency exceeds 300 Hz, the COD value and viscosity decrease slowly. Studies have shown that increasing the pulse frequency can promote the formation of active substances, such as ozone, and improve the energy yield of the system. Considering the processing effect and cost, the pulse frequency is 300 Hz.

### 2.3. Treatment of Hydroxypropyl-Guar Wastewater by DBD–LTP Coupled with AgIn_5_S_8_/g-C_3_N_4_

**Figure 3:**
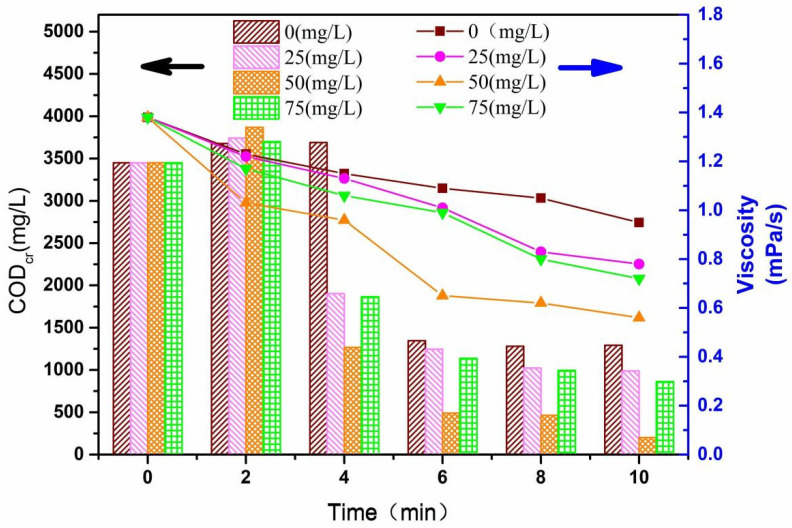
Effect of photocatalyst dosage on COD and viscosity of hydroxypropyl-guar wastewater by coupling process (voltage: 120 V; ventilation: 12 L/min; discharge time: 8 min; pulse frequency: 300 Hz; pH: 4).

The effect of the AgIn_5_S_8_/g-C_3_N_4_ nano-photocatalyst dosage in photocatalytic-coupled DBD–LTP on the COD and viscosity of treated wastewater is shown in Figure 3. It can be seen from the chart that compared with the plasma technology without the photocatalyst, the COD and viscosity of the wastewater treated by the three groups of experiments with the photocatalyst are greatly reduced. With the coupling treatment, in addition to the active substances produced by the original plasma system, the added photocatalyst also fully absorbs the light and energy in the system to produce substances with catalytic degradation activity, thus promoting the degradation of hydroxypropyl guar. It can be seen from the figure that in the first two minutes of the coupling treatment, the COD of wastewater increased to varying degrees, which also proved that the addition of the photocatalyst promoted the further degradation of refractory groups in guar gum. In addition, in the experimental group without the photocatalyst, the property change in wastewater slowed down after treatment for about 8 min, while the time of slowing down the property change in wastewater after treatment was advanced after adding the catalyst. When the dosage of the catalyst was 50 mg/L, this time was advanced to about 4~6 min. Therefore, the coupling treatment not only enhanced the degradation degree of hydroxypropyl guar, but also improved its degradation efficiency. By comparing the results of the two groups of experiments (Figure 2c and Figure 3) with the same discharge parameters, it can be seen that the time required when the COD_Cr_ dropped to the lowest value (1296 mg/L) is 8 min in the DBD test, while in the coupling experiment, the time required when the wastewater COD_Cr_ reached about 1296 mg/L is only 4 min. The comparative analysis can prove that under the same discharge conditions, the photocatalyst absorbed the ultraviolet light in the coupling process and played a photocatalytic role, and improved the degradation efficiency of hydroxypropyl guar. Therefore, the coupling technology can effectively improve the energy utilization rate of the discharge system and enhance the degradation efficiency of polymer organic matter.

### 2.4. Detection of Active Substances in the Liquid Phase

**Figure 4:**
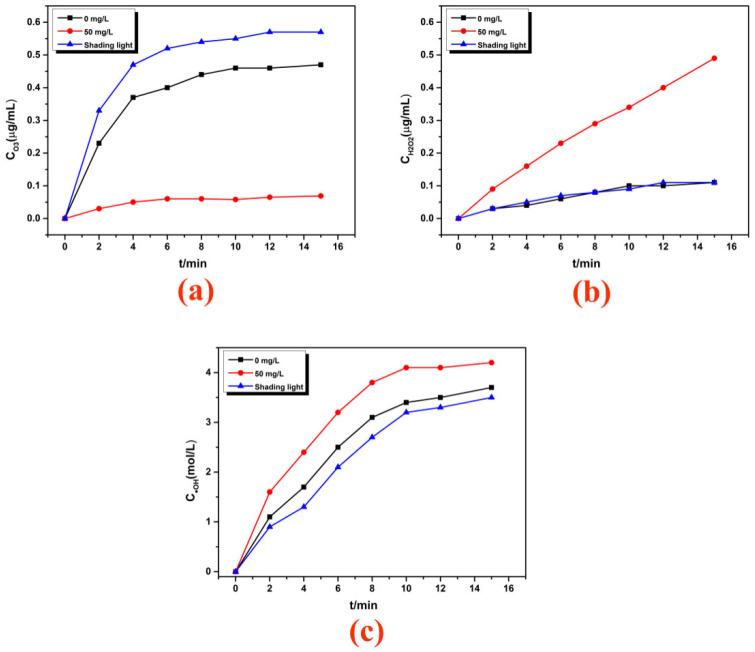
(**a**) The concentration of aqueous O_3_; (**b**) the concentration of H_2_O_2_; (**c**) the concentration of ·OH radicals. (Without pollution in solution; the concentration of AgIn_5_S_8_/g-C_3_N_4_: 50 mg/L or 0 mg/L; voltage: 120 V; ventilation: 12 L/min; discharge time: 8 min; pulse frequency: 300 Hz; pH: 4).

Many studies showed that high-voltage discharge could stimulate photocatalytic reactions to achieve the coordinated treatment of organic pollutants, which ultimately came down to active substances. Usually, in the majority of the active substances produced by the DBD discharge system, O_3_ accounts for the vast majority, which also contains ·OH and H_2_O_2_, and the three active substances transform with each other. However, O_3_ has the oxidation ability and can oxidize and degrade organic substances, but its oxidation ability is not as good as ·OH, and it cannot produce an effect on some refractory organic matter. Therefore, it was necessary to analyze the changes of the active substances in the DBD system under different conditions to explore the mechanism of the enhanced degradation of hydroxypropyl guar in the coupled treatment process. The results are shown in Figure 4.

O_3_ + hv → ·O_2_ + ·O (1)

·O + H_2_O → 2·OH (2)

2·OH → 2H_2_O_2_ (3)

H_2_O_2_ + hv → 2·OH (4)

**Figure 5:**
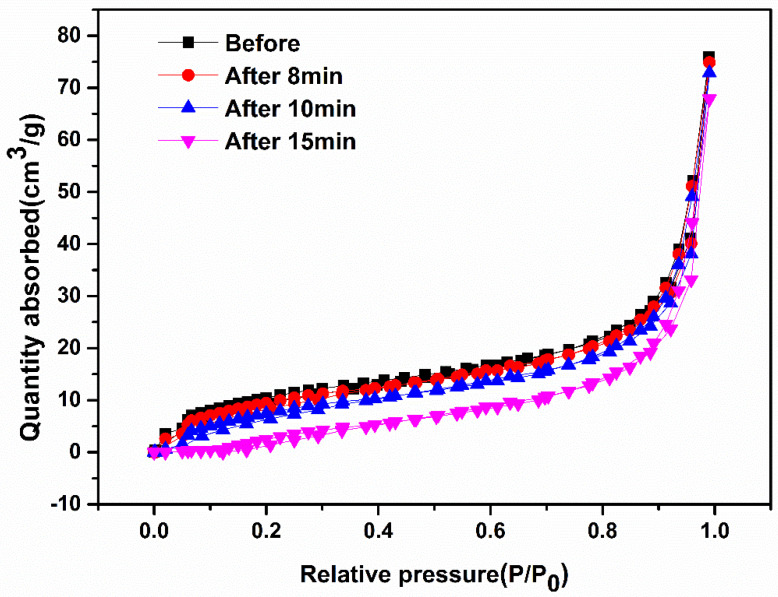
N_2_ absorption-desorption isotherms of the AgIn_5_S_8_/g-C_3_N_4_ composite.

The increase in O_3_ concentration under light shading conditions was due to the inhibition of photolysis of O_3_ under the absence of light conditions, which also reduced the concentration of H_2_O_2_ and ·OH (Equations (1)–(4)). It also verified that the discharge radiation was an important factor of the pollutant treatment; so adding the photocatalyst to the solution could make full use of the discharge radiation and achieve the effect of organic matter degradation synergies. According to Figure 4, when the photocatalyst AgIn_5_S_8_/g-C_3_N_4_ was added to the discharge system, the concentration of O_3_ decreased significantly, while the concentration of H_2_O_2_ and ·OH increased. One reason was that electron–hole pairs produced in the photocatalytic reactions reacted with O_3_ and H_2_O_2_, respectively (Equations (5)–(9)). On the other hand, it may be that the addition of the photocatalyst affected the solubility of O_3_ and reduced its concentration significantly. As is also seen from Figure 5, the rate of H_2_O_2_ generation was reduced, probably because the AgIn_5_S_8_/g-C_3_N_4_ andNO_3^−^_ produced during the discharge consumed ·OH. Based on the analysis of the active species in the system and the results of previous studies, it can be speculated that the effective degradation of hydroxypropyl guar in the coupling process was mainly attributed to two aspects. One is the direct oxidation and degradation of hydroxypropyl guar by the active substances (such as electrons, ions, ·OH) produced by the DBD system discharge; the other is the photocatalytic degradation by the active substances produced by the interaction transformation between photogenic electrons/holes produced by photocatalysts absorbing UV light and the active species in the system.

AgIn_5_S_8_/g-C_3_N_4_ + hv/visible → e^−^ + h^+^ (5)

e^−^ + O_3_ → ·O_2_ + ·O + e^−^ (6)

h+ + H_2_O → H+ + ·OH (7)

e^−^ + 2H_2_O_2_ → ·OH + OH^−^ (8)

h^+^ + H_2_O_2_ → H^+^ + HO_2_· (9)

### 2.5. Effect of DBD Discharge on AgIn_5_S_8_/g-C_3_N_4_

**Figure 6:**
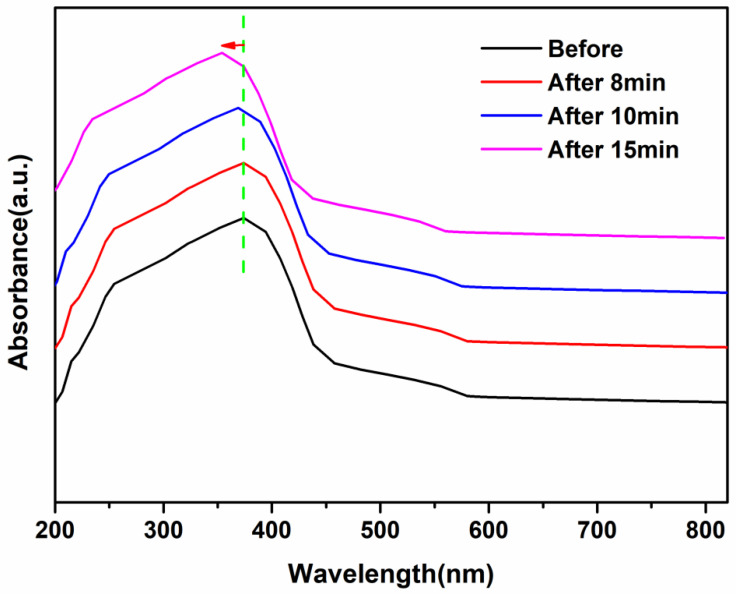
The UV/Vis DRS spectra of the AgIn_5_S_8_/g-C_3_N_4_ composite.

It is well known that the physical and chemical effects of high-voltage discharges can directly or indirectly treat material surfaces and degrade pollutants in wastewater and waste gas. However, the study of the application of low-temperature plasma in pollutant treatment is limited to the production and utilization of active substances, and the indirect effect of its physical effects has been ignored or has not been deeply studied. It has been shown that the discharge of the DBD system will affect the specific surface area and pore structure of g-C_3_N_4_ material and affect its catalytic activity. However, the interaction of the DBD system discharge and photocatalytic materials has not been deeply studied, such as the detailed action mechanism between the high-voltage discharge and photocatalyst. BET analysis and UV/Vis DRS spectra were used to investigate the effects of the physical structure and optical properties of AgIn_5_S_8_/g-C_3_N_4_ materials before and after discharge of the DBD system. Results of the experiments are shown in Figure 5 and Figure 6.

Figure 5 shows the nitrogen adsorption-desorption isotherm of AgIn_5_S_8_/g-C_3_N_4_ material before and after discharge. As can be seen from Figure 5, a lag ring was formed before and after discharge, indicating slit medium holes were produced on the material. When the discharge time was 8 min, the adsorption amount of the material was almost the same as the raw material; when the discharge time continued to be extended, the adsorption amount of the material was reduced but not obvious. This showed that the DBD air discharge (milder) did not significantly change the specific surface area and pore volume of the material. This is likely because of the unselective physical effects of low-temperature plasma techniques, unlike the selective destruction of strongly oxidized solutions. Moreover, the oxidation of DBD discharge often follows the physical effect of the discharge. When the material is destroyed and forms a defect, the active substance will be immediately oxidized and defective, which may produce chemical functional groups that may cause side effects on the surface of AgIn_5_S_8_/g-C_3_N_4_.

The optical properties of the AgIn_5_S_8_/g-C_3_N_4_ samples at different discharge times are shown in Figure 6. As can be seen, when the discharge time was short, the light absorption band edge of the sample was not affected by the low-temperature plasma. This can be explained by the lack of defects, such as carbon and nitrogen vacancies, on the sample surface. Related studies show that the g-C_3_N_4_ sample incorporated by oxygen has no effect on the valence band but changes the edge of the absorption band [39,40]. As can be seen from Figure 6, there is a slight band edge blue shift in the discharge time at 15 min, possibly because of the long discharge time, the physical and chemical effects of the discharge promoting the generation of nanoparticles, and the quantum constraint effect also appeared. The method of adjusting the microstructure and bandgap by changing the discharge treatment time is similar to changing the pyrolysis time. Due to the low oxygen content on the sample surface and the slight layer destruction, the microstructure and band gap changes were not obvious.

## 3. Materials and Methods

### 3.1. Materials

All the synthetic raw materials of the composite photocatalyst (AgIn_5_S_8_/g-C_3_N_4_) were analytical grade and purchased from Sigma-Aldrich. Additionally, reagents and solvents related to the water quality analysis and hydroxypropyl guar were purchased from Chongqing Chuandong Chemical Industry Co., Ltd., Chongqing, China. All reagents were used in the study without further purification. The details of the main reagents are shown in Table 1.

### 3.2. DBD–LTP System

**Figure 7:**
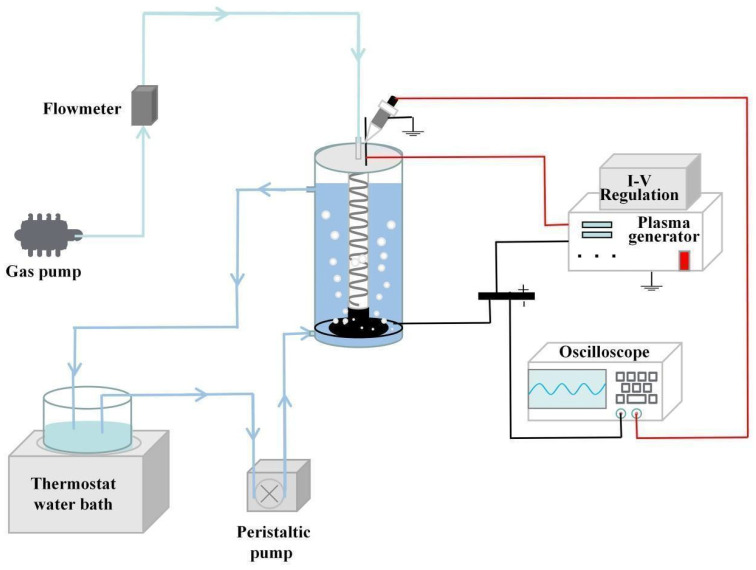
The DBD–LTP system.

The DBD–LTP system is shown in Figure 7. This system consists of the aeration system, wastewater cooling circulation system, medium blocking discharge reactor, and high-voltage AC power supply.

The wastewater stored in the constant temperature tank entered from the inlet to the wastewater treatment container (effective volume of 1.6 L) under the action of the peristaltic pump, while the air entered the quartz pipe through the air pump and flow meter. During operation, the plasma produced in the quartz tube came out of the aeration head in the form of microbubbles under the impetus of air and made contact with the pollutants in the wastewater flowing outside the pipe. Meanwhile, the ultraviolet and visible light produced by the discharge entered through the quartz pipe into the wastewater. Pollutants were effectively decomposed under the strong oxidation of active substances and ultraviolet light.

### 3.3. Preparation of AgIn_5_S_8_/g-C_3_N_4_

AgIn_5_S_8_ nanoparticles were combined with g-C_3_N_4_ by the chemical wet method. The specific operation was as follows: 1.0 g g-C_3_N_4_ was ultrasonically dispersed in ethanol aqueous solution (v%, 66%) for 1 h. Then, AgIn_5_S_8_ nanoparticles were added with the mass fraction of g-C_3_N_4_ and was controlled at 15~40%. The mixture was then continuously stirred for 12 h. The precipitate was centrifuged and dried overnight in a vacuum drying oven at 70 °C. The dried product was the target photocatalyst.

### 3.4. Photocatalytic Activity and Mechanism of AgIn_5_S_8_/g-C_3_N_4_

A total of 50 mL hydroxypropyl-guar wastewater (0.3 wt.%) was added into the photo-reactor, and then, predetermined quality AgIn_5_S_8_/g-C_3_N_4_ photocatalysts were added to perform the photocatalytic degradation experiments. Before light, a dark adsorption-desorption reaction (10 min) was performed to achieve a balance between the catalyst and the hydroxypropyl-guar solution. After a certain light time, 2 mL of the degradation solution was centrifuged, and the supernatant was taken for COD_cr_ and viscosity measurement.

### 3.5. Treatment of Hydroxypropyl-Guar Wastewater by DBD–LTP

The hydroxypropyl-guar wastewater (0.3 wt.%) was pumped into the reactor at a set flow rate, and the DBD–LTP reactor parameters (voltage and pulse frequency) and aeration strength were adjusted to investigate the impact of the discharge time, voltage, pulse frequency, and aeration strength on the degradation of hydroxypropyl guar.

### 3.6. Treatment of Hydroxypropyl-Guar Wastewater by DBD–LTP Coupled with AgIn_5_S_8_/g-C_3_N_4_

Firstly, a quantitative photocatalyst was added into the hydroxypropyl-guar wastewater (0.3 wt.%). Then, the wastewater was pumped into the reactor at a set flow rate, and the DBD–LTP reactor parameters (voltage and pulse frequency) and aeration strength were adjusted to the appropriate values. After the reaction, the COD and viscosity of the wastewater at the outlet were determined to investigate the effect of the photocatalyst dosage on hydroxypropyl-guar degradation.

### 3.7. Detection of Active Substances in the Liquid Phase of DBD–LTP Sysytem

Pure water or pure water containing a 50 mg/L photocatalyst was pumped into the plasma reactor at a flow rate of 0.2 L/min, while adjusting the reactor parameters (voltage of 120 V and pulse frequency of 300 Hz) and aeration strength (2.8 m^3^/m^3^·min). Salicylic acid was used as a ·OH scavenger, and the total contents of 2, 5-dHBA and 2, 3-dHB generated from the reaction of salicylic acid and ·OH in solution was determined by liquid chromatography to determine the concentration of the ·OH produced by the system discharge. The concentrations of H_2_O_2_ and O_3_ in the water were measured by the titanium salt colorimetric method and sodium blue disulfonate fade spectrophotometry, respectively. In addition, the discharge quartz tube was shaded with tin foil before operation, and pure water was used as the treatment medium; the concentration of active substances were measured under the same operating conditions described above.

## 4. Conclusions

The effective degradation treatment of fracturing wastewater has always been the bottleneck in the development of low-permeability oil and gas fields. In view of this, this paper studied the degradation treatment of photocatalytic and low-temperature plasma technology for simulated fracturing wastewater containing hydroxypropyl guar, and further explored the coupling of the two processes for the degradation of simulated wastewater. The results showed that hydroxypropyl-guar wastewater could be degraded to a certain extent by either photocatalytic technology or plasma technology, and the COD and viscosity of the treated wastewater under two single-technique optimal conditions were 781 mg·L^−^^1^, 0.79 mPa·s^−^^1^ and 1296 mg·L^−^^1^, 1.01 mPa·s^−^^1^, respectively. Furthermore, the effective coupling of AgIn_5_S_8_/gC_3_N_4_ photocatalysis and DBD–LTP not only enhanced the degradation degree of hydroxypropyl guar but also improved its degradation efficiency. Under the optimal conditions of coupling treatment, the hydroxypropyl-guar wastewater achieved the effect of a single treatment within 6 min, and the COD and viscosity of the treated wastewater reduced to below 490 mg·L^−^^1^ and 0.65 mPa·s^−^^1^, respectively. Accordingly, this paper proposed a new idea of treating oilfield fracturing wastewater. The research results could not only enrich the theoretical system of organic fracturing wastewater degradation, but also guide the practical application of the coupling technology in the environmental protection of low permeability oilfields.

## Figures and Tables

**Table 1:** The details of the main reagents.

Reagents	Molecular Formula	CAS	Purity Grade
Melamine	C_3_H_6_N_6_	108-78-1	AR
Dilute nitric acid ^①^	HNO_3_	7697-37-2	AR
Silver nitrate	AgNO_3_	7761-88-8	AR
Indium nitrate	In(NO_3_)_3_·4H_2_O	13770-61-1	AR
Ethylene glycol	(CH_2_OH)_2_	107-21-1	AR
Thioacetamide	CH_3_CSNH_2_	62-55-5	AR
Ethyl Alcohol	CH_3_CH_2_OH	64-17-5	AR
Hydroxypropyl guar	C_3_H_8_O_2_·x	39421-75-5	AR

^①^ The concentration of dilute nitric acid: 6 mol/L.

## Data Availability

The data presented in this study are available in the article.
